# The Highly Accurate Interatomic Potential of CsPbBr_3_ Perovskite with Temperature Dependence on the Structure and Thermal Properties

**DOI:** 10.3390/ma16052043

**Published:** 2023-03-01

**Authors:** Qianyu You, Shun Gu, Xiaofan Gou

**Affiliations:** College of Mechanics and Materials, Hohai University, Nanjing 211100, China

**Keywords:** CsPbBr_3_, potential, bond valence, elastic constants, thermal conductivity

## Abstract

CsPbBr_3_ perovskite has excellent optoelectronic properties and many important application prospects in solar cells, photodetectors, high-energy radiation detectors and other fields. For this kind of perovskite structure, to theoretically predict its macroscopic properties through molecular dynamic (MD) simulations, a highly accurate interatomic potential is first necessary. In this article, a new classical interatomic potential for CsPbBr_3_ was developed within the framework of the bond-valence (BV) theory. The optimized parameters of the BV model were calculated through first-principle and intelligent optimization algorithms. Calculated lattice parameters and elastic constants for the isobaric–isothermal ensemble (*NPT*) by our model are in accordance with the experimental data within a reasonable error and have a higher accuracy than the traditional Born–Mayer (BM) model. In our potential model, the temperature dependence of CsPbBr_3_ structural properties, such as radial distribution functions and interatomic bond lengths, was calculated. Moreover, the temperature-driven phase transition was found, and the phase transition temperature was close to the experimental value. The thermal conductivities of different crystal phases were further calculated, which agreed with the experimental data. All these comparative studies proved that the proposed atomic bond potential is highly accurate, and thus, by using this interatomic potential, the structural stability and mechanical and thermal properties of pure inorganic halide and mixed halide perovskites can be effectively predicted.

## 1. Introduction

Due to its unique photovoltaic characteristics, halide perovskite not only has broad prospects in the photovoltaic power generation industry, but also has potential technological applications in other aspects [1,2,3,4]. Hence, a growing stream of research involving halide perovskite has been both experimentally and theoretically conducted by technologists. It has been reported that the solar cell efficiency for halide perovskite has grown rapidly from 2.2% in 2006 to 25.2% at present [5,6,7].

As a superior photoelectric application material, premium lead perovskite (CsPbBr_3_), which is a typical ABC_3_ perovskite with a cubic phase (space group pm-3m), has been intensively studied. In the crystal structure of CsPbBr_3_, Pb^2+^ occupies the lattice center, Cs^+^ occupies the vertex and Br^2−^ is located in the plane center [8,9]. Since photoelectric properties are closely related to the phase transition and structural stability, CsPbBr_3_ has been brought into research, and experiments have shown that the structural phase of CsPbBr_3_ changes from an orthorhombic system to a tetragonal system at 373 K and to a cubic system at 403 K [10,11] due to the Jahn–Teller effect. In addition, given the high experimental cost and considerable computational time required, atomic simulations with little loss of accuracy provide a valid measure for exploring the internal mechanism of structural phase transformations. Therefore, to better characterize the long-term effect of temperature on the structural stability and phase transformation of CsPbBr_3_ over a long period, we find that classical molecular dynamics (CMD) is more effective than density functional theory (DFT) [12,13,14]. Density functional theory (DFT) is one of the most reasonable physical theories to describe cell potential energy. DFT describes the lattice parameters of the dense solid system, and the binding energy and elastic modulus are consistent with the experimental measurements. Although the first-principles method has achieved success in studying the structure and the electronic and optical properties of CsPbBr_3_, the huge difficulties in studying the continuous temperature properties have promoted the development of atomic potentials and Hamiltonian potentials.

The Born–Mayer potential (referred to as the traditional BM potential in this paper) is the most common in molecular dynamics simulations of CsPbBr_3_ perovskites [15,16,17]. The calculation results show that the traditional BM can accurately characterize the overall structural change in the unit cell during phase transition, but when the phase transition and internal PbBr_3_ octahedral structure are distorted at the same time, the traditional BM potential cannot accurately describe the composite structural distortion, leading to the phase transition not being predicted. This is because traditional BM potentials only consider pairwise interactions. Therefore, there is an urgent need for the development of a more targeted potential function.

In this paper, the BV potential function model is employed to calculate the most intuitive structural transformation: the lattice parameter distortion and the length of the Pb-Br bond in the crystal cell. In addition to the structural and mechanical properties, the thermal properties (including thermal conductivity, thermal expansion coefficient and specific heat capacity) of CsPbBr_3_ play a major role in industrial applications, which is a key feature of solar cell heat transfer. To characterize heat transfer and resistance, the equilibrium molecular dynamics (EMD) method is adopted to calculate the coefficient of thermal conductivity (*κ*), which agrees reasonably well with previous experimental results [18,19].

## 2. Methodology

### 2.1. Potential Model

A classical interatomic potential for CsPbBr_3_ is established within the framework of the bond-valence theory and the bond-valence vector-conservation principle [20,21,22]. According to the conservation rule, the total bond valence of each atom i tends to be equivalent to its initial atomic valence $V_{i,0}$ in the ground-state structure. The actual valence $V_{i}$ of atom i is the sum of the ionic valence $v_{ij}$, as Equation (1) shows. Moreover, $v_{ij}$ can be modeled by using the inverse power–law correlation between bond length and ionic valence given in Equation (2), where $R_{0,ij}$ and $C_{ij}$ are Brown’s empirical parameters. Equations (1) and (2) are as follows

(1) $$V_{i}=\sum_{j\neq i}v_{ij}$$

(2) $$v_{ij}={\left(\frac{R_{0,ij}}{r_{ij}}\right)}^{C_{ij}}$$

Based on Brown’s bond-valence theory, the interatomic potential of the developed bond-valence model is expressed by the following equations:

(3) $$E_{tot}=E_{c}+E_{r}+E_{BV}+E_{BVV}$$

in which

(4) $$E_{c}=\sum_{i\neq j}k{\frac{q_{i}q_{j}}{r_{ij}}}_{}$$

(5) $$E_{r}={\sum_{i\neq j}\left(\frac{A_{ij}}{r_{ij}}\right)}^{12}$$

(6) $$E_{BV}={\sum B_{i}\left(V_{i}-V_{i,0}\right)}^{2}$$

(7) $$E_{BV}{}_{V}={\sum C_{i}\left(U_{i}-U_{i,0}\right)}^{2}$$

where the total energy $E_{tot}$ consists of non-bond potentials ($E_{c}$, $E_{r}$) and bond potentials ($E_{BV}$, $E_{BVV}$), respectively. Among them, $E_{c}$ is Coulomb energy, Coulomb constant is 14.4 eV/Å, and $q_{i}$ is the ionic charge. $E_{r}$ is the short-range repulsive Lennard-Jones energy and $A_{ij}$ is a short-range repulsion parameter between different types of atoms (Pb-Cs, Pb-Br, Cs-Br and Br-Br). $E_{BV}$ is the bond-valence energy, $B_{i}$ and $C_{i}$ are the proportional factors of energy unit and $V_{i}$ is the valence electron of the *i*th atom computed by the bond-valence theory.

$E_{BVV}$ is the bond-valence vector energy, representing the measure of local symmetry breaking. $U_{i,0}$ is the initial key valence vector value and $U_{i}$ is the absolute value of the sum of $v_{ij}$ vectors, calculated via the following equation [23,24,25]:

(8) $$U_{i}=\left|\sum_{i\neq j}v_{ij}\right|$$

**Figure 1:**
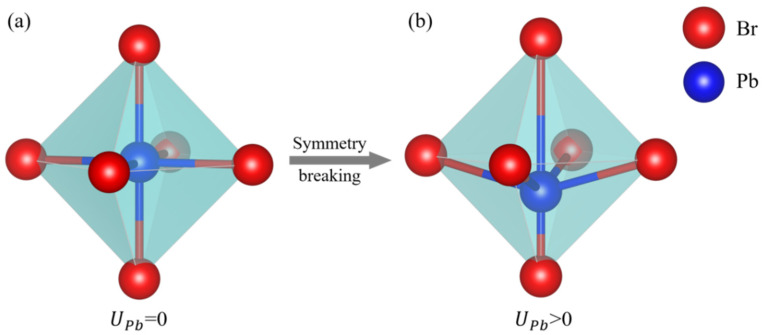
Different structures presented by CsPbBr_3_. (**a**) $U_{Pb}$ = 0 when Pb-Br bonds are in perfect symmetry distribution; (**b**) $U_{Pb}$ > 0 when the cell structure of CsPbBr_3_ is asymmetric.

As shown in Figure 1, when CsPbBr_3_ exhibits a quintessential perovskites cubic structure, $U_{Pb}$ = 0. Instead, when no structural symmetry is present, $U_{Pb}$ > 0. Therefore, in the BV potential model of CsPbBr_3_, the parameters we need to fit are $A_{ij}$, $B_{i}$, $C_{i}$ (i = Cs, Pb, Br), and the specific fitting method will be introduced in the next section. 

### 2.2. Method of BV Potential Parameters

**Figure 2:**
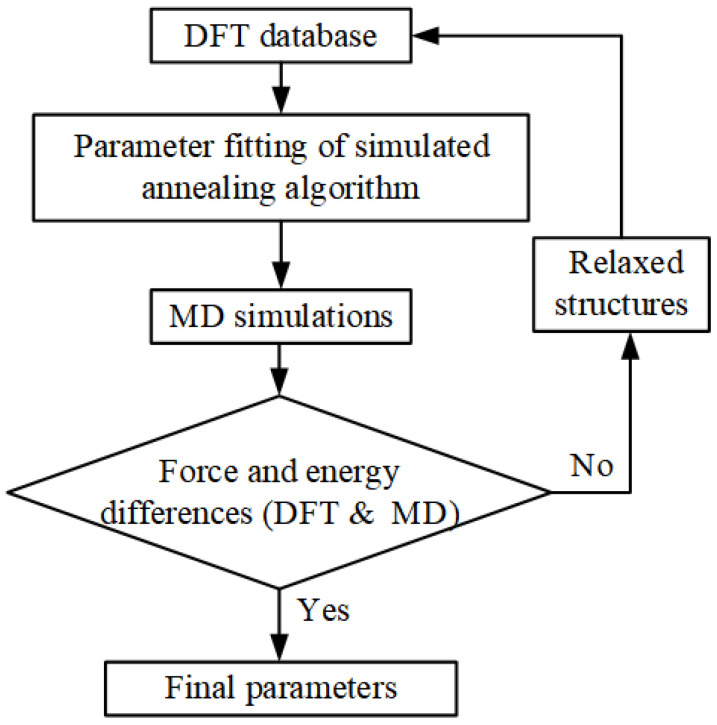
Parameter fitting protocol performed using the Simulation Optimization Algorithm.

The fitting of the potential parameters $A_{ij}$, $B_{i}$, $C_{i}$ (i = Cs, Pb, Br) is performed by minimizing the force and energy differences between the DFT calculation and the BV model. The complete parameters fitting flow chart is shown in Figure 2. 

In order to obtain as much structural data as possible to fully fit the BV potential parameters, the vc-md calculation method (molecular dynamics method under the first-principles framework) is used. The initial parameters of CsPbBr_3_ crystal lattices originate from experimental values given in Refs. [26,27]. According to scale, the initial lattice parameters in equal proportion range from −10% to 10%, the multiple equilibrium structures calculated are selected as the initial database on the condition that cell size change and atomic movement are allowed. In addition, Quantum-Espresso (QE) is a first-principles computing software package based on DFT. With this software package, 260 CsPbBr_3_ structures were obtained, including orthogonal structures, tetragonal structures and basic cubic structures. The pseudopotentials in this work were generated using the Perdew-Burke-Ernzerhof (PBE) [28] version of Generalized Gradient Approximation (GGA) to estimate the exchange correlation. The convergence tests for cutoff energy and *k*-point mesh samplings were fulfilled one by one. A plane wave cutoff energy of 46 Ry and Brillouin zone sampled by a 12 × 12 × 12 Monkhorst–Pack *k*-point mesh was employed for energy and force calculations. Then, the annealing algorithm (SA) was optimized for the linear combination of the BV potential function model and DFT calculation of structural energy *E* and atomic force *F*: ∑(|Edef−E(qi,Aij,Bi,Ci)|+|Fdef-F(qi,Aij,Bi,Ci)|). The following formula can obtain the atomic force from the potential energy of the BV model: F=−dE/dr.

Then, the iterative conditions were analyzed. To ensure the rationality of the energy and atomic force differences calculated by our BV model, we selected 30 comparative samples with different temperatures and structures for calculations. The next step is to bring the parameters into molecular dynamics calculations. If the energy and force differences between MD simulation and DFT results are large, the new structure obtained by MD relaxation will continue to be input into the initial database, and finally, include the balanced multi-state and high-energy unbalanced structure. After several cycles, the energy and force between MD and DFT are approached. The optimized BV potential parameters are shown in Table 1.

## 3. Results and Discussion

### 3.1. Lattice Parameters and Elastic Constants

According to the above fitting process, the BV potential function parameters of CsPbBr_3_ were obtained and their reliability was verified by comparing them with the average atomic energy difference of DFT. However, the accuracy of the description of specific structural characteristics still needs to be further verified. The Lattice parameters, as the important structural parameters of crystals, are often employed to characterize molecular system changes and cell phase transitions. Moreover, the elastic constants that express the stress–strain relationship within the stress range are employed to evaluate the mechanical stability of CsPbBr_3_. In this section, the validity of the optimized BV potential parameters was tested via molecular dynamic (MD) simulations with the lattice parameters and elastic constants fixed to theoretical and experimental values, as implemented in the Linear Atomic/Molecular Massive Parallel Simulator (LAMMPS) [29] open-source software package.

CsPbBr_3_ undergoes the orthorhombic–tetragonal–cubic phase transitions at room temperature, 373 K and 403 K [27], respectively. Therefore, the lattice parameters and elastic constants of the three different structures were calculated by the MD simulation. Then, we carried out *NPT* ensembles using a 7 × 7 × 6 orthorhombic supercell with 5880 atoms at 300 K, a 7 × 6 × 6 tetragonal supercell with 5040 atoms at 380 K and a 6 × 6 × 6 cubic phase supercell with 4320 atoms at 600 K. The temperature was controlled by a Nose–Hoover thermostat and the pressure was maintained at 1 atm by a Parrinello–Rahman thermostat. The force field cutoff distance and time-step were set as 8 angstroms and 2 fs, respectively. There was a total of 120,000 running steps. 

To compare the correctness of the lattice parameters and elastic constants calculated by MD simulation, Birch–Murnaghan’s equation of state [30] and Born stability criteria [31] were employed to obtain the parameters of all three compounds by DFT method. The detailed calculation method is as follows.

**Figure 3:**
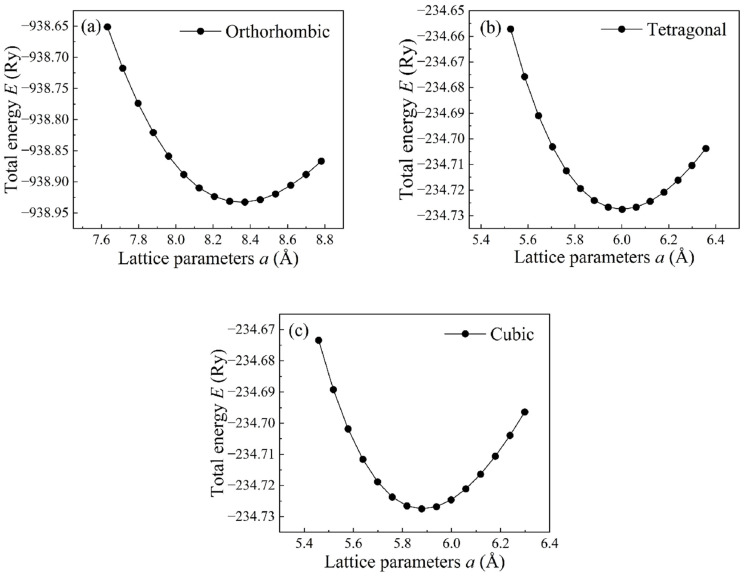
Variation of total energy as a function of lattice parameters for CsPbBr_3_. (**a**) orthorhombic structure; (**b**) tetragonal structure; (**c**) cubic structure.

Birch–Murnaghan’s equation of state [30], as shown in Equation (10), was employed to evaluate the lattice parameters of CsPbBr_3_ by fitting the unit-cell energy *E* versus the unit-cell volume *V* of each compound. In this equation, we have

(9) $$E(V)=E_{0}+\frac{B_{0}V}{B_{0}^{\prime }}\left(\frac{{\left(V_{0}/V\right)}^{B_{0}^{\prime }}}{B_{0}^{\prime }-1}+1\right)-\frac{B_{0}V_{0}}{B_{0}^{\prime }-1}$$

where *E*_0_ and *V*_0_ are the equilibrium unit-cell energy and volume, respectively. *B*_0_ is the bulk modulus and $B_{0}^{\prime }$ is its pressure derivative, which ranges from 3.3 to 5.5. These calculations were performed by the DFT method that was implemented in the QE package. The convergence threshold of total energy is 1.0 × 10^−5^ Ry and that on forces is 1.0 × 10^−4^ Ry/Bohr for ionic minimization. To achieve an accurate convergence, the Monkhorst–Pack schemes with 12 × 12 × 8, 16 × 16 × 16 and 16 × 16 × 16 *k*-mesh were employed for the calculation of orthorhombic, tetragonal and cubic structures of bulk CsPbBr_3_, respectively. In the optimization procedure, the calculated lattice parameters *a* converted from volumes *V* were plotted against corresponding energies *E*. The fitting curves for all three compounds are plotted in Figure 3. The optimum lattice parameters correspond to the minimum energy of the unit cell, and then, lattice parameters *a* in the ground state were evaluated.

On the other hand, elastic constants were obtained using ElaStic software [32], which provided a tool to figure out elastic constants from total energy and stress calculations with QE codes. In this study, we preferred total energy for elastic constant calculations. The values of elastic coefficients (*C*_11_, *C*_12_, *C*_13_, *C*_22_, *C*_23_, *C*_33_, *C*_44_, *C*_55_ and *C*_66_) need to be determined using the Projector Augmented Wave (PAW) with the Perdew–Burke–Ernzerhof (PBE) [28] approach. The essential Born stability criteria [31] are listed below for the three different structures of CsPbBr_3_: (i). *C*_11_ > 0, *C*_22_ > 0, *C*_33_ > 0, *C*_44_ > 0, *C*_55_ > 0, *C*_66_ > 0; (ii). [*C*_11_ + *C*_22_ + *C*_33_ + 2 (*C*_12_ + *C*_13_ + *C*_23_)] > 0; (iii). (*C*_11_ + *C*_22_ − 2*C*_12_) > 0; (iv). (*C*_11_ + *C*_33_ − 2*C*_13_) > 0; (v). (*C*_22_ + *C*_33_ − 2*C*_23_) > 0. To evaluate the mechanical behavior of CsPbBr_3_, it is necessary to determine the elastic modulus under applied stress. According to the Hill approximations, the bulk modulus (*B*) and Shear modulus (*G*) can be obtained from the values of elastic coefficients, as shown in the following formulas [33,34]

(10) $$B=\frac{C_{11}+2C_{12}}{3}$$

(11) $$G=\frac{\left[\frac{C_{11}-C_{12}+3C_{44}}{5}\right]+\left[\frac{5C_{44}\left(C_{11}-C_{12}\right)}{4C_{44}+3\left(C_{11}-C_{12}\right)}\right]}{2}$$

Combined with Equations (10) and (11), the values of Young’s modulus (*E*) are calculated as follows

(12) $$E=\frac{9BG}{3B+G}$$

The comparison of the lattice parameters and elastic constants of CsPbBr_3_ given by the MD simulation with experimental values, LDA, DFT calculation and the BV model potential, is shown in Table 2. It can be seen that the lattice parameters calculated by the DFT method are close to the experimental values (error less than 3%), indicating good reliability of data sources in an initial database. When CsPbBr_3_ presents the cubic phase under high temperature, the lattice parameters calculated by the BV potential are in good agreement (error less than 2%) with the experimental values. When CsPbBr3 is orthogonal at room temperature, the accuracy of the BV model is higher than that of other models and the error is within 1%. On the other hand, the bulk modulus is calculated to implement the characterization of the material’s ability to resist compression in Table 2, which is in good agreement with the reported value. This suggests that CsPbBr_3_ is strong enough to return to its original volume after applied stress is removed. Moreover, the strength of CsPbBr_3_ against plastic deformation and cracking or breakage is, respectively, demonstrated by the shear modulus and Young’s modulus, which is consistent with theoretical (DFT method) and experimental results (error less than 20%). The Pugh ratio (*B*/*G*), calculated from the ratio of the bulk modulus to the shear modulus, can be employed to evaluate the ductility/brittleness, and a Pugh ratio of more than 1.75 indicates that CsPbBr_3_ is ductile. Through the above analysis, the mechanical and structural stability of CsPbBr_3_ at different temperatures can be proven to be excellent.

Based on the analysis of the lattice parameters and elastic constants of CsPbBr_3_, we conclude that Jahn–Teller distortion is the most complex when CsPbBr_3_ is in the orthogonal phase, and there is a large deviation from the characteristic of a typical *Pnma* space group. At this time, LDA cannot accurately reflect the distortion caused by the electronic structure level. The BV potential function model constructed for the perovskite structure is more accurate to describe the deformation and distortion of a regular octahedron, which is a major factor contributing to the phase transformation.

**Figure 4:**
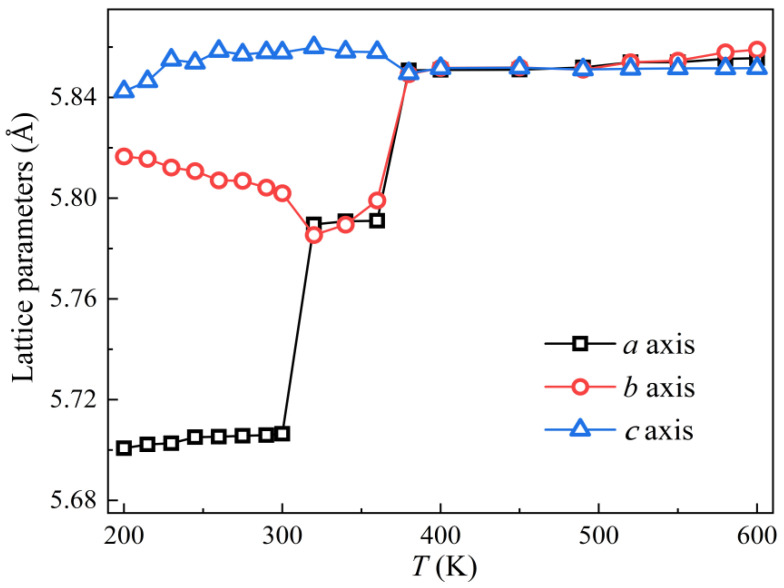
Changes in lattice parameters of cubic CsPbBr_3_ along three crystallographic axes at different temperatures using the BV model.

In order to comprehensively observe the structural distortion trend of CsPbBr_3_ during phase transformation, an 11 × 11 × 11 cubic CsPbBr_3_ supercell of 6655 atoms was constructed and relaxed under the action of BV potential function. The simulated environment was the *NPT* ensemble with a pressure of 1 atm and a temperature range of 200 K~600 K. The time step was set to 2fs and a total of 100,000 steps were run. The results after relaxation are shown in Figure 4. The equilibrium structure under multiple temperature fields was simulated, and the values of *a*, *b* and *c* axes were significantly different. From Figure 4, CsPbBr_3_ cells present an orthonormal phase at 200 K. With the increase in temperature, the *a* and *c* axes tend to be equal, and CsPbBr_3_ cells transform into a tetragonal phase at 310 K. At 380 K, the triaxial values are almost equal and the cells transform into cubic phase.

Compared with BM potential [17], our BV potential function model is more accurate because of the failure to show the structural transition with temperature for the BM potential function. In the MD simulation, it can be seen that the phase transition temperature is underestimated. This underestimation has been observed in other previous DFT fitting simulations [37] due to systematic errors in the exchange-correlation function used for field optimization. 

### 3.2. Bond Lengths

For CsPbBr_3_ perovskite, with the increase in system temperature, the crystal structure will undergo orthogonal–tetragonal–cubic phase transformation. This process is accompanied by Jahn–Teller structural deformation and torsion of the PbBr_3_ octahedron, which is the main reason for the complex phase transformation of CsPbBr_3_. In the PbBr_3_ octahedron, the deformation is mainly reflected in the stretching of the Pb-Br ionic bond length along the *a*, *b* and *c* axes. By processing the position information of the structure atoms obtained from the relaxation, the changes in the ionic bond length in the structural distortion can be described.

The radial distribution function (RDF) is the ratio of the local density around an ion to the bulk density when determining ion coordinates [38,39]. It is employed to describe the density distribution of particles around the central particle in the multi-particle system. RDF curves are generally spectral where a peak denotes the presence of a particle and the first peak denotes the location of the nearest particle. We calculate the bond length of Pb-Br and perform RDF analysis at a cutoff distance of 8 angstroms. The BV model is used to calculate the average Pb-Br bond length in the *ac* plane and at the *b*-axis of the orthogonal structure at 300 K, in the *ac* plane and at the *b*-axis of the tetragonal structure at 380 K, and in the cubic structure at 600 K, respectively.

**Figure 5:**
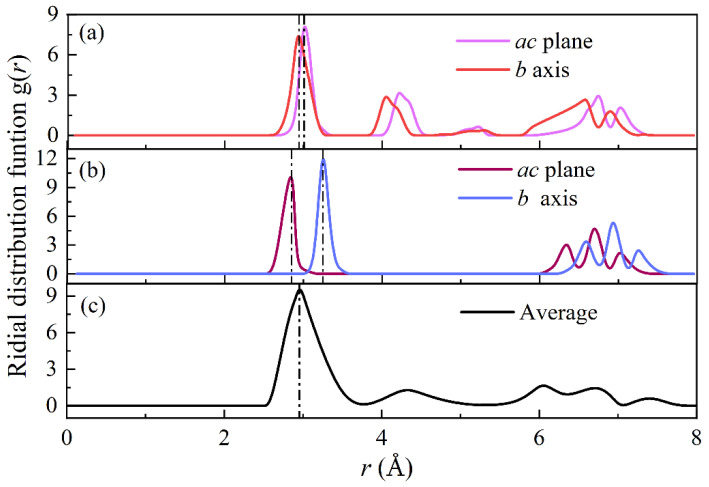
Length of three axes of Pb-Br bond in the CsPbBr_3_ structure: (**a**) orthorhombic at 300 K; (**b**) Tetragonal at 380 K; (**c**) cubic at 600 K.

As can be seen from the RDF curve in Figure 5 and the Pb-Br bond length in Table 3, when CsPbBr_3_ presents an orthogonal structure, the change in the Pb-Br bond length along the *ac* plane and *b* axis suggests the occurrence of phase transformation and octahedral distortion, which is in good agreement with the experimental observation results [14,39] within a 2% error. When CsPbBr_3_ presents a cubic symmetric phase, the three axial Pb-Br bonds are consistent, and the error between the calculated value with BV potential function and the experimental value is less than 0.2%, indicating that the phase distortion in the expansion of the Pb-Br ionic bond is simpler during the phase transformation of CsPbBr_3_.

### 3.3. Thermal Conductivity

It is important for well-designed hybrid perovskite-based energy-conversion devices [40] to understand the thermal transport in the CsPbBr_3_. The knowledge of thermodynamic properties of CsPbBr_3_ has been limited by the difficulty of attaining an experimental measurement of thermal conductivity below room temperature [41,42]. Therefore, the theoretical prediction of thermal conductivity for CsPbBr_3_ is quite necessary. 

Molecular dynamics is often used as an effective theory to predict the thermal conductivity of materials. The commonly used methods are equilibrium molecular dynamics (EMD) and non-equilibrium molecular dynamics (NEMD). However, both methods require a high degree of precision, and slight deviations can make a difference in orders of magnitude. In this section, the thermal conductivity of CsPbBr_3_ is calculated in LAMMPS using the EMD method to verify the applicability and accuracy of the BV potential function.

The EMD method is based on the Green-Kubo formula for thermal conductivity κ. This equation relates the transport index of the non-equilibrium structure to the fluctuations of the physical quantities of the equilibrium structure. The transport index is defined as the integral of the correlation time of the autocorrelation function, shown in Equation (13) as

(13) $$\kappa_{uv}(t)=\frac{V}{kBT^{2}}\int_{0}^{t}dt\prime C_{uv}(t^{\prime })$$

where $\kappa_{uv}$ and $C_{uv}$ denote the thermal conductivity tensor and heat current autocorrelation function (HCACF), respectively. It is assumed that CsPbBr_3_ has isotropic characteristics in different phase states, and the 4 × 3 × 3 orthogonal supercell of 720 atoms, 4 × 2 × 3 tetragonal supercell of 480 atoms and 3 × 3 × 3 cubic supercell of 540 atoms are established as the calculation model. The initial equilibrium temperature was set at 300 K and the ambient pressure was set at 1 atm (NVT ensemble). In the potential field, the potential field cutoff energy is 8 Å, the time step is set as 0.04 fs and the balance steps are 150,000, with a total time of 6 ps.

**Figure 6:**
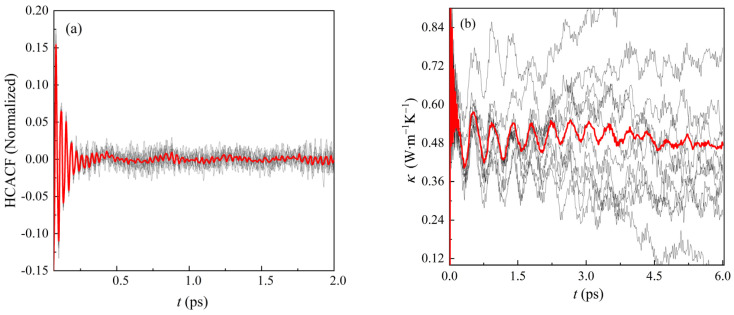
Thermal conductivity of CsPbBr_3_ at 300 K calculated by the BV model. (**a**) Heat current autocorrelation function; (**b**) thermal conductivity *κ*.

According to the orthogonal CsPbBr_3_ model at 300 K, heat current autocorrelation function and thermal conductivity were calculated, as shown in Figure 6. Here, the red line represents the average values of ten simulated calculations. Heat current autocorrelation function flow becomes close to zero as time passes, which is consistent with the theoretical trend. The thermal conductivity curve changes with simulation time, and the value of thermal conductivity tends to be stable at last, which is 0.497 W‧m^−1^ K^−1^. The thermal conductivity of CsPbBr_3_ can be experimentally measured as 0.42 ± 0.04 W‧m^−1^ K^−1^ at room temperature [18,19]. By contrast, the error between simulation values obtained by BV potential and experiment values is within 30%. The BV potential function calculation results are better than the DFT calculation results [43].

**Figure 7:**
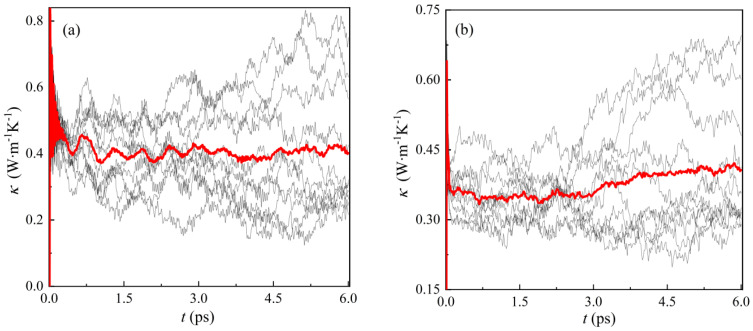
(**a**) Thermal conductivity *κ* of tetragonal structure at 380 K; (**b**) thermal conductivity *κ* for cubic structure at 600 K.

As shown in Figure 7, the thermal conductivity of tetragonal phase at 380 K and cubic phase at 600 K are 0.412 W‧m^−1^ K^−1^ and 0.395 W‧m^−1^ K^−1^, respectively. The red line represents the average values of ten simulated calculations. Unfortunately, we did not find experimental data on thermal conductivity at high temperatures for comparison. However, we compared the thermal conductivity of materials with a similar structure to CsPbBr_3_, shown in Table 4 (the unit is W‧m^−1^ K^−1^). It can be seen that the thermal conductivity of CsPbBr_3_ gradually decreases with increasing temperature, which is in accordance with the trends of CsPbI_3_ and CsSnI_3_. Moreover, the BV model results are closer to the experimental values than the DFT results for CsPbBr_3_ [43], and the BV model results are quite close to the experimental values of CsPbI_3_ and CsSnI_3_ [44] within a 10% error range. Similar to DFT simulation methods, the accuracy of thermal conductivity *κ* in the MD simulation largely depends on the accuracy of the potential function. To obtain more accurate BV potential, there are more factors to be considered in the process of parameter fitting. For example, thermal conductivity calculations are continuously added to the database when fitting parameters. Another method is to add a thermal conductivity influence term to the BV potential model, which can be expressed as a function of temperature. The BV potential function provides a new idea for the thermal conductivity simulation of CsPbBr_3_.

## 4. Conclusions

In this article, for CsPbBr3 perovskites, the new BV potential function was developed based on I. D. Brown’s bond-valence theory, in which the potential parameters were obtained by minimizing the energy and force differences between the DFT method and the BV model. The calculated lattice parameters, elastic constants, bond lengths and thermodynamic properties of CsPbBr_3_ by the MD method verified the reliability and accuracy of potential parameters for the BV model through a comparison with the experimental data or DFT values. The final results and conclusions were as follows. We used the BV potential energy model to study the orthorhombic, tetragonal and cubic structures of CsPbBr_3_. Compared with LDA, the BV potential had higher accuracy in describing the lattice parameters, elastic constants and Pb-Br bond lengths, as well as their changing trends in different phase states, and the thermal conductivity calculated by the BV potential was very close to the experimental value. This new BV potential model could be further used for effectively predicting the structural stability and the mechanical and thermal properties of pure inorganic halide and mixed halide perovskites.

## Figures and Tables

**Table 1:** BV model potential parameters of CsPbBr_3_.

$A_{ij}\;(\text{Å})$							
	Cs	Pb	Br	$B_{i}$	$C_{i}(\mathrm{eV})$	$V_{i,0}$	$U_{i,0}$
Cs	2.304	2.585	2.539	1.448	2.094	1.00	0.151
Pb	-	2.390	2.495	1.497	1.932	2.00	0.080
Br	-	-	2.455	1.714	1.909	−1.00	0.396

**Table 2:** Lattice parameters and elastic constants of orthogonal, tetragonal and cubic structures calculated through BV model, DFT method, LDA and experiment.

Structure		Lattice Parameters		Bulk Modulus	Shear Modulus	Young’s Modulus	Pugh Ratio
	*a* (Å)	*b*(Å)	*c* (Å)	*B* (GPa)	*G* (GPa)	*E* (GPa)	*B*/*G*
**Orthorhombic**							
Experimental	^a^ 8.250	^a^ 11.700	^a^ 8.212	^d^ 15.50	^d^ 5.93	^d^ 15.80	^d^ 2.63
LDA	^b^ 8.561	^b^ 12.190	^b^ 8.51	^e^ 18.45	^e^ 7.90	^e^ 20.74	^e^ 2.34
DFT	8.404	11.971	8.355	18.29	7.14	18.95	2.56
BV model	8.292	11.600	8.291	14.95	6.90	17.94	2.17
Error	0.51%	0.85%	0.96%	3.55%	16.36%	13.54%	17.49%
**Tetragonal**							
Experimental	^c^ 5.864	^c^ 5.864	^c^ 5.811	-	-	-	-
LDA	^b^ 5.772	^b^ 5.772	^b^ 5.828	-	-	-	-
DFT	5.991	5.991	5.952	21.56	7.92	21.17	2.72
BV model	5.776	5.771	5.804	18.16	7.43	19.61	2.44
Error	1.50%	1.59%	0.12%	-	-	-	-
**Cubic**							
Experimental	^c^ 5.874	^c^ 5.874	^c^ 5.874	-	-	-	-
LDA	^b^ 5.772	^b^ 5.772	^b^ 5.772	^f^ 19.43	^f^ 8.31	^f^ 21.69	^f^ 2.34
DFT	5.878	5.878	5.878	18.37	6.33	17.03	2.90
BV model	5.802	5.802	5.802	18.13	6.91	18.27	2.62
Error	1.22%	1.22%	1.22%	-	-	-	-

Orthogonal, Tetragonal and Cubicis repressent crystal structures of CsPbBr_3_. Ref. [27] ^a^, Ref. [13] ^b^, Ref. [28] ^c^, Ref. [35] ^d^, Ref. [17] ^e^, Ref. [36] ^f^.

**Table 3:** Pb-Br bond length calculated by DFT, BV model and comparison with experimental results.

Structure	Experiment	DFT	BV Model/Error
**Orthorhombic**			
*ac* plane	2.931	2.906	2.945/0.48%
*b* axis	2.977	2.943	3.006/0.82%
**Tetragonal**			
*ac* plane	2.821	2.910	2.851/1.06%
*b* axis	3.310	3.350	3.25/1.81%
**Cubic**			
Average	2.952	2.935	2.955/0.10%

Orthogonal, Tetragonal and Cubicis repressent crystal structures of CsPbBr_3_.

**Table 4:** The thermal conductivity calculated by the BV model and comparison with materials with a similar structure to CsPbBr_3_.

		300 K	380 K	600 K
CsPbBr_3_	Experimental	0.42	-	-
	BV model	0.497	0.412	0.395
	DFT	~0.80	~0.76	~0.70
CsPbI_3_	Experimental	0.45	-	-
	DFT	~0.38	~0.37	~0.35
CsSnI_3_	Experimental	0.38	-	-
	DFT	~0.63	~0.55	~0.45

## Data Availability

The data presented in this study are available on request from the corresponding author. The data are not publicly available due to privacy.

## References

[1] Gao W., Zhang Z., Xu R., Chan E.M., Yuan G., Liu J. (2022). Development and Prospects of Halide Perovskite Single Crystal Films. Adv. Electron. Mater..

[2] Dey A., Ye J., De A., Debroye E., Ha S.K., Bladt E., Kshirsagar A.S., Wang Z., Yin J., Wang Y. (2021). State of the Art and Prospects for Halide Perovskite Nanocrystals. ACS Nano.

[3] Wang R., Huang T., Xue J., Tong J., Zhu K., Yang Y. (2021). Prospects for metal halide perovskite-based tandem solar cells. Nat. Photon..

[4] Haque M., Kee S., Villalva D.R., Ong W.L., Baran D. (2020). Halide perovskites: Thermal transport and prospects for thermoelectricity. Adv. Sci..

[5] Deng J., Li J., Yang Z., Wang M. (2019). All-inorganic lead halide perovskites: A promising choice for photovoltaics and detectors. J. Mater. Chem. C.

[6] Gao Y., Wu Y., Lu H., Chen C., Liu Y., Bai X., Yang L., Yu W.W., Dai Q., Zhang Y. (2019). CsPbBr3 perovskite nanoparticles as additive for environmentally stable perovskite solar cells with 20.46% efficiency. Nano Energy.

[7] Ma X., Yang L., Zheng S. (2020). All-Inorganic perovskite solar cells: Status and future. Prog. Chem..

[8] Brennan M.C., Kuno M., Rouvimov S. (2019). Crystal Structure of Individual CsPbBr_3_ Perovskite Nanocubes. Inorg. Chem..

[9] Dastidar S., Hawley C.J., Dillon A.D., Gutierrez-Perez A.D., Spanier J.E., Fafarman A.T. (2017). Quantitative Phase-Change Thermodynamics and Metastability of Perovskite-Phase Cesium Lead Iodide. J. Phys. Chem. Lett..

[10] Nagal V., Kumar V., Kumar R., Singh K., Khosla A., Ahmad R., Hafiz A.K. (2021). CsPbBr3 nanoplatelets: Synthesis and understanding of ultraviolet light-induced structural phase change and luminescence degradation. ECS J. Solid State Sci. Technol..

[11] Svirskas Š., Balčiūnas S., Šimėnas M., Usevičius G., Kinka M., Velička M., Kubicki D., Castillo M.E., Karabanov A., Shvartsman V.V. (2020). Phase transitions, screening and dielectric response of CsPbBr3. J. Mater. Chem. A.

[12] Ahmad M., Rehman G., Ali L., Shafiq M., Iqbal R., Ahmad R., Khan T., Jalali-Asadabadi S., Maqbool M., Ahmad I. (2017). Structural, electronic and optical properties of CsPbX3 (X=Cl, Br, I) for energy storage and hybrid solar cell applications. J. Alloys Compd..

[13] Ghaithan H.M., Alahmed Z.A., Qaid S.M.H., Hezam M., Aldwayyan A.S. (2020). Density functional study of cubic, tetragonal, and orthorhombic CsPbBr3 Perovskite. ACS Omega.

[14] López C.A., Abia C., Alvarez-Galván M.C., Hong B.-K., Martínez-Huerta M.V., Serrano-Sánchez F., Carrascoso F., Castellanos-Gómez A., Fernández-Díaz M.T., Alonso J.A. (2020). Crystal Structure Features of CsPbBr_3_ Perovskite Prepared by Mechanochemical Synthesis. ACS Omega.

[15] Singh H., Singh A., Indu B.D. (2016). The Born-Mayer-Huggins potential in high temperature superconductors. Mod. Phys. Lett. B.

[16] Almishal S.S.I., Rashwan O. (2020). New accurate molecular dynamics potential function to model the phase transformation ofcesium lead triiodide perovskite. RSC Adv..

[17] Balestra S.R., Vicent-Luna J.M., Calero S., Tao S., Anta J.A. (2020). Efficient modelling of ion structure and dynamics in inorganic metal halide perovskites. J. Mater. Chem. A.

[18] Haeger T., Wilmes M., Heiderhoff R., Riedl T. (2019). Simultaneous Mapping of Thermal Conductivity, Thermal Diffusivity, and Volumetric Heat Capacity of Halide Perovskite Thin Films: A Novel Nanoscopic Thermal Measurement Technique. J. Phys. Chem. Lett..

[19] Lee W., Li H., Wong A., Zhang D., Lai M., Yu Y., Kong Q., Lin E., Urban J.J., Grossman J.C. (2017). Ultralow thermal conductivity in all-inorganic halide perovskites. Proc. Natl. Acad. Sci. USA.

[20] Brown I.D. (1978). Bond valences—A simple structural model for inorganic chemistry. Chem. Soc. Rev..

[21] Brown I.D. (2009). Recent Developments in the Methods and Applications of the Bond Valence Model. Chem. Rev..

[22] Brown I.D. (2017). What is the best way to determine bond-valence parameters?. IUCrJ.

[23] Anbalagan K., Thomas T. (2018). Size-dependent disproportionation (in~ 2–20 nm regime) and hybrid Bond Valence derived interatomic potentials for BaTaO2N. Appl. Nanosci..

[24] Liu S., Grinberg I., Takenaka H. (2016). Reinterpretation of the bond-valence model with bond-order formalism: An improved bond-valence-based interatomic potential for PbTiO3. Phys. Rev. B.

[25] Shin Y.-H., Cooper V.R., Grinberg I., Rappe A.M. (2005). Development of a bond-valence molecular-dynamics model for complex oxides. Phys. Rev. B.

[26] Atourki L., Vega E., Mollar M., Marí B., Kirou H., Bouabid K., Ihlal A. (2017). Impact of iodide substitution on the physical propertiesand stability of cesium lead halide perovskite thin films CsPbBr3-xIx (0 ≤ x ≤ 1). J. Alloys Compd..

[27] Moller C.K. (1959). The structure of perovskite-like caesium plumbotrihalides. Mater. Fys. Medd. Dan. Vid. Selsk..

[28] Giannozzi P., Baroni S., Bonini N., Calandra M., Car R., Cavazzoni C., Ceresoli D., Chiarotti G.L., Cococcioni M., Dabo I. (2009). QUANTUM ESPRESSO: A modular and open-source software project for quantum simulations of materials. J. Phys. Condens. Matter.

[29] Michael T.H., Yong Z., Edward J.M. (2019). PyLAT: Python LAMMPS Analysis Tools. J. Chem. Inf. Model..

[30] Nagaoka Y., Hills-Kimball K., Tan R., Li R., Wang Z., Chen O. (2017). Nanocube Superlattices of Cesium Lead Bromide Perovskites and Pressure-Induced Phase Transformations at Atomic and Mesoscale Levels. Adv. Mater..

[31] Zheng C., Rubel O. (2017). Ionization Energy as a Stability Criterion for Halide Perovskites. J. Phys. Chem. C.

[32] Golesorkhtabar R., Pavone P., Spitaler J., Puschnig P., Draxl C. (2013). ElaStic: A tool for calculating second-order elastic constants from first principles. Comput. Phys. Commun..

[33] Azzouz L., Halit M., Sidoumou M., Charifi Z., Allal A., Bouchenafa M., Baaziz H. (2019). Electronic structure, elastic and optical properties of AEuS2 (A = Na, K, Rb, and Cs) ternary sulfides: First-principles study. Phys. Status Solidi..

[34] Tan J., Li Y., Ji G. (2012). Elastic constants and bulk modulus of semiconductors: Performance of plane-wave pseudopotential and local-density-approximation density functional theory. Comput. Mater. Sci..

[35] Rakita Y., Cohen S.R., Kedem N.K., Hodes G., Cahen D. (2015). Mechanical properties of APbX3 (A = Cs or CH3NH3; X = I or Br) perovskite single crystals. MRS Commun..

[36] Maphoto R.I., Ngoepe P., Masedi M., Morukuladi M., Malatji K.T. (2022). First-Principle Study of CsPbBr3 and CsPbI3 Perovskite Solar Cells. ECS J. Solid State Sci. Technol..

[37] Jinnouchi R., Lahnsteiner J., Karsai F., Kresse G., Bokdam M. (2019). Phase Transitions of Hybrid Perovskites Simulated by Machine-Learning Force Fields Trained on the Fly with Bayesian Inference. Phys. Rev. Lett..

[38] Haider N., Hossain F. (2018). CSV2RDF: Generating RDF data from CSV file using semantic web technologies. J. Theor. Inf. Technol..

[39] Stoumpos C.C., Malliakas C.D., Peters J.A., Liu Z., Sebastian M., Im J., Chasapis T.C., Wibowo A.C., Chung D.Y., Freeman A.J. (2013). Crystal Growth of the Perovskite Semiconductor CsPbBr_3_: A New Material for High-Energy Radiation Detection. Cryst. Growth Des..

[40] Chang X., Li W., Zhu L., Liu H., Geng H., Xiang S., Liu J., Chen H. (2016). Carbon-Based CsPbBr_3_ Perovskite Solar Cells: All-Ambient Processes and High Thermal Stability. ACS Appl. Mater. Interfaces.

[41] Pisoni A., Jacimovic J., Barisic O.S., Spina M., Gaál R., Forró L., Horváth E. (2014). Ultra-low thermal conductivity in organic-inorganic hybrid. J. Phys. Chem. Lett..

[42] Kovalsky A., Wang L., Marek G.T., Burda C., Dyck J.S. (2017). Thermal conductivity of CH3NH3PbI3 and CsPbI3: Measuring the effect of the methylammonium ion on phonon scattering. J. Phys.Chem. C.

[43] Tadano T., Saidi W.A. (2022). First-Principles Phonon Quasiparticle Theory Applied to a Strongly Anharmonic Halide Perovskite. Phys. Rev. Lett..

[44] Kawano S., Tadano T., Iikubo S. (2021). Effect of Halogen Ions on the Low Thermal Conductivity of Cesium Halide Perovskite. J. Phys. Chem. C.

